# Soil analysis in discussions of agricultural feasibility for ancient civilizations: A critical review and reanalysis of the data and debate from Chaco Canyon, New Mexico

**DOI:** 10.1371/journal.pone.0198290

**Published:** 2018-06-14

**Authors:** Jon-Paul P. McCool, Samantha G. Fladd, Vernon L. Scarborough, Stephen Plog, Nicholas P. Dunning, Lewis A. Owen, Adam S. Watson, Katelyn J. Bishop, Brooke E. Crowley, Elizabeth A. Haussner, Kenneth B. Tankersley, David Lentz, Christopher Carr, Jessica L. Thress

**Affiliations:** 1 Department of Geography and GIS, University of Cincinnati, Cincinnati, Ohio, United States of America; 2 Department of Geography & Meteorology, Valparaiso University, Valparaiso, Indiana, United States of America; 3 Department of Anthropology, University of Cincinnati, Cincinnati, Ohio, United States of America; 4 School of Anthropology, University of Arizona, Tucson, Arizona, United States of America; 5 Department of Anthropology, University of Virginia, Charlottesville, Virginia, United States of America; 6 Department of Geology, University of Cincinnati, Cincinnati, Ohio, United States of America; 7 Division of Anthropology, American Museum of Natural History, New York, New York, United States of America; 8 Department of Anthropology, University of California, Los Angeles, California, United States of America; 9 Department of Biology, University of Cincinnati, Cincinnati, Ohio, United States of America; New York State Museum, UNITED STATES

## Abstract

Questions about how archaeological populations obtained basic food supplies are often difficult to answer. The application of specialist techniques from non-archaeological fields typically expands our knowledge base, but can be detrimental to cultural interpretations if employed incorrectly, resulting in problematic datasets and erroneous conclusions not easily caught by the recipient archaeological community. One area where this problem has failed to find resolution is Chaco Canyon, New Mexico, the center of one of the New World’s most vibrant ancient civilizations. Discussions of agricultural feasibility and its impact on local population levels at Chaco Canyon have been heavily influenced by studies of soil salinity. A number of researchers have argued that salinized soils severely limited local agricultural production, instead suggesting food was imported from distant sources, specifically the Chuska Mountains. A careful reassessment of existing salinity data as measured by electrical conductivity reveals critical errors in data conversion and presentation that have misrepresented the character of the area’s soil and its potential impact on crops. We combine all available electrical conductivity data, including our own, and apply multiple established conversion methods in order to estimate soil salinity values and evaluate their relationship to agricultural productivity potential. Our results show that Chacoan soils display the same salinity ranges and spatial variability as soils in other documented, productive fields in semi-arid areas. Additionally, the proposed large-scale importation of food from the Chuska Mountains region has serious social implications that have not been thoroughly explored. We consider these factors and conclude that the high cost and extreme inflexibility of such a system, in combination with material evidence for local agriculture within Chaco Canyon, make this scenario highly unlikely. Both the soil salinity and archaeological data suggest that there is no justification for precluding the practice of local agriculture within Chaco Canyon.

## Introduction

Whether it has been through the investigation of individual remains (e.g. [1]) or entire landscapes (e.g. [2]), growth in interdisciplinary studies within archaeology has unequivocally increased our knowledge of past cultures across the globe. By opening up the range of approaches to understanding past peoples, existing research questions can be answered in new ways and new questions can be incorporated into archaeological discussions. However, this opportunity can also be perilous as errors in analyses can result in misleading conclusions that become part of the canon for particular regions. An archaeologist working with the implications of these results may not have the requisite training to assess the highly specialized methods employed by the initial study, while scientists trained in the utilized methodology may not be exposed to the results presented in the archaeological literature or aware of critical contextual information. In this environment, it becomes increasingly important for archaeological scientists and interdisciplinary teams to critically evaluate existing work, particularly when this work challenges archaeological expectations. We review and assess the application of a common and relatively simple method for assessing soil salinity from archaeological contexts in Chaco Culture National Historical Park, located in northwestern New Mexico. While archaeologists have long debated the implications of these studies, the results have largely remained unchallenged, as most archaeologists working in the region did not have the soil science background necessary to assess the data. Ultimately, a thorough reassessment of the original data, as well as the addition of data collected by a recent project, does not substantiate the original interpretations, instead suggesting agriculture was feasible for Chaco residents. This case study at a World Heritage Site, one of the most important archaeological regions in North America [3], highlights both the benefits and the dangers of interdisciplinary studies in archaeological research.

The 800–1130 C.E. occupation of Chaco Canyon was characterized by the construction and occupation of large multistoried pueblos, known as great houses (e.g. [3–6]). These structures contained an impressive array of rare and nonlocal materials, including turquoise, macaws, cacao, copper bells, and shells (e.g. [7–11]). Additionally, dozens of small sites, resembling in size and form other pueblos in the region, were occupied contemporaneously within the canyon (e.g., [12,13]). The presence of great house communities, known as outliers, throughout the northern Southwest indicated that the “Chaco Phenomenon” [14] emanated far beyond the canyon boundaries, but the extent and nature of interaction between outlier communities and the canyon proper varied (e.g. [15–18]). Relationships among residents of the great houses, small sites, and many of the outliers remain poorly understood, although these interactions are a crucial variable in sociopolitical reconstructions [19].

Despite over a century of research in the canyon, the demography, social structure, and economic base of the Chaco people remain topics of significant disagreement among researchers. Several sociopolitical systems have been proposed, including: a pilgrimage and/or redistribution center, a “location of high devotion,” a kingdom, and a hierarchy based on house societies [5,20–23]. In particular, the pilgrimage or redistribution model, which posited that the canyon served as a ritual center to which individuals from the surrounding region travelled annually for a combination of religious, social, and economic benefits (e.g. [5, 24–29]), is intertwined with two controversial assumptions deriving from ideas about local environmental conditions and the Chacoan economy. First, Chaco Canyon was believed to be a marginal environment—an “unlikely setting” for substantial human occupation ([3], p9)—leading to debates over population size. Some scholars have suggested that the great houses were sparsely inhabited ceremonial centers that housed only a small priestly population and served primarily as storage space and temporary shelter for visitors [5,30,31]. Second, claims of extremely limiting environmental conditions implied that the resident population required support through the regular importation of food, such as maize and meat (e.g. [5,32–36]). Scheduled pilgrimage events are hypothesized to have facilitated large-scale transport of food to the canyon. Despite mounting research findings that question the central premises of the pilgrimage model (e.g. [37–39]), Chaco’s purported environmental limitations continue to influence estimations of population size and food availability and are generally used to support the idea of a “vacant ceremonial center” (c.f. [40]), although some researchers incorporate food importation into hierarchical models that assume a sizable local population as well (e.g. [21]).

**Fig 1:**
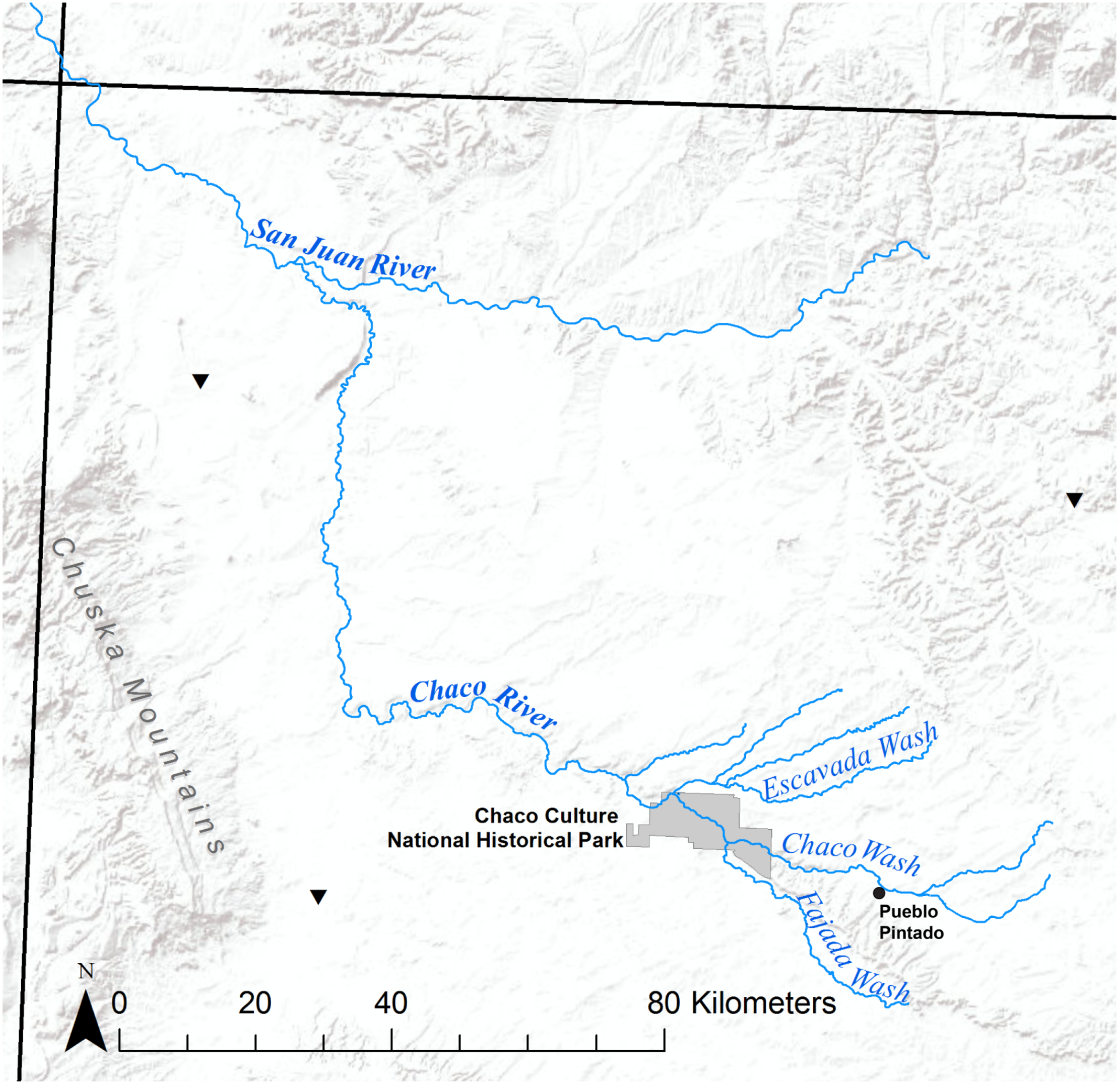
General location of Chaco Canyon Cultural Historical Park in relation to the Chuska Mountains. Four Corners is in upper left corner of figure at intersection of black state lines. Only selected major drainages contributing to or near Chaco Wash are represented. Black triangles are the three closest pedons to Chaco Canyon that have been sampled by the USDA.

The ability to successfully practice agriculture was particularly important for Puebloan occupants of the Southwest. It is estimated that maize accounted for about 80% of people’s diet based on likely caloric consumption during Chaco’s occupation [41–45]. While several factors influence estimates of agricultural productivity, measures of soil salinity, or the amount of salt within soil, have been integral to arguments for minimal potential maize agriculture within the canyon for over a decade (e.g. [46,47]). Recently, two reassessments of soil salinity have questioned its effects on maize agriculture, coming to vastly different conclusions. Tankersley et al. [48] tested samples from modern Pueblo fields and locations likely to be ancient fields within Chaco Canyon and did not find salinity levels that would have prohibited maize production. Based on these results, the authors proposed that maize agriculture within the canyon, in most years, could have supported most or all of a sizable resident population. Alternately, Benson [33] reiterated his long-standing proposition that the majority of the canyon floor would have been unsuitable for maize due to high salt levels within the soils. Instead, he posited that the Chuska Mountains likely supplied the majority of the maize required to support Chaco residents (see Fig 1 for location). Although Chaco Canyon acquired many nonlocal resources demonstrating their far-reaching ties and influence (e.g. [10,11,49–52]), the postulated large-scale importation of maize, a staple of the Pueblo diet, would have greatly increased the quantity and regularity of material transport, suggesting intensive contact with, and reliance on, outside groups.

The Tankersley [53] and Benson [33] papers are the most recent examples of a long-standing debate about the agricultural potential of Chaco Canyon (e.g. [36,54–56]). In this paper, we review the Chaco salinity debate in detail to assess these studies and their broader implications for our understanding of the potential agricultural productivity and social and political organization of the canyon. First, we provide an introduction to soil salinity and its effects on plants, followed by a detailed discussion of the methods by which it is measured. This is followed by a review of prior studies of soil salinity within Chaco Canyon, noting where inappropriate methods have produced questionable results necessitating a reassessment of the local agricultural productivity potential of the region. Finally, we consider the social implications of large-scale maize importation to the canyon and briefly present other lines of evidence in support of local agricultural production.

## Review of soil salinity & electrical conductivity measurement

The amount of salt in a soil can prevent plants from taking up water, even if water is in physical contact with their roots. As such, soil salinity is an important component of agricultural success. To give non-specialists an introduction to the topic, we present a brief overview of the nature of chemical salts and the formation of a salt solution, the physics of water uptake from such a solution, and the common methodologies used to assess the salinity, or salt concentration, in a solution.

### Salts

In chemistry, a salt is a neutral ionic compound composed of balanced anions and cations that can dissolve in water to create free moving ions in solution. Salts dissolve into solution when the bonds holding a solid salt together are overcome by the pull of polarized water molecules. This means that one type of salt (e.g., NaCl) may dissolve more easily than another (e.g., CaSO_4_^.^ 2H_2_O) due to differing bond strengths. Water cannot dissolve an infinite amount of salt: as more salt enters into solution within a given amount of water, eventually no further salt ions can be pulled away from the solid. As the volume of water is reduced (by evaporation, percolation, or plant uptake), salt will precipitate back into solid form. In arid region soils, salts may be variously present in either solid form or in solution depending on soil moisture levels.

### Soil water & root uptake

Plants use three different methods for drawing water into their roots (see [57]), but all are based fundamentally on osmosis. The movement of water out of soil into roots requires the existence of higher osmotic pressure within soil water than within a plant’s roots, which will spur water molecules to move out of the soil and into the root. Salinity lowers osmotic potential within soil water and, therefore, reduces the amount of water available to plant roots. Although the precise combination of causes and effects is debated [58], the greater the amount of salts in a soil, the lower its osmotic potential, and the greater the hold the soil will have on water relative to a plant root. Thus, soil salinity can cause plants to wilt through a lack of water, even if water is physically present within the soil.

### Electrical conductivity & assessing salinity

To measure how much salt is present within a soil, researchers utilize the fact that ions in a solution, such as those from salt dissociation in soil water, conduct an electric current relative to their concentration. Thus, a measure of electrical conductivity (EC) via a conductivity bridge–two metal probes in a solution in which electrical current flows from one to the other–can be used as a proxy for ion concentration with higher conductivity indicative of higher ion concentration (Note A in S1 Notes). Since ions that carry the electrical charge must be free to move, conductivity measurements cannot be made on a solid soil sample and must be made on a soil-water mixture or solution extracted from such a mixture. However, from this simple step, a large number of complicating factors arise that can result in the misapplication of the technique.

The most common approach is the measurement of EC based on a combination of pure, often deionized, water with a soil sample in a laboratory setting. The standard comparative method is the creation of a saturated paste (a mixture of soil and water that is at a point that the sample may flow slightly and does not stick to its container), vacuum extraction of the water from the saturated sample, and a conductivity measurement on that extract (see procedure 4F2 in [59]). This method and its results, abbreviated using EC_e_, are considered the established, preferred procedure in agricultural studies. Either because determining EC_e_ is a more involved process or because it requires a substantial amount of sample (250–300 grams), many researchers, and even agricultural laboratories, utilize a set ratio dilution of soil and water. Published practices vary in the soil to water ratios used (1:1, 1:2, 1:2.5, or 1:5) and the length of time the solution is given to equilibrate (e.g., 1 hour [60], 2 hours [61], 8 hours [62], and 24 hours for procedure 4F1b1 [59]). Further variation is introduced based on whether the measurement is taken on the soil-water mixture or on the soil-water solution after being extracted under vacuum, whether dried bulk sample or ground bulk samples are used, and the temperature of the measured solution. Each variable can change the resulting EC measurement and complicate, if not preclude, comparison among results.

The greatest concerns for inter-study comparability are variations in soil to water ratios and equilibration time (see Table 1). The concentration of ions in solution decreases with increasing dilution of a soil sample, resulting in a lower measured EC (e.g. [63]). In practice, EC does not have a linear relationship with concentration due to a number of factors, including: Ion specific charge differences in soils dominated by specific salt species (e.g. [64] Table 2 differences in EC_1:1_ to EC_e_ conversion due to specific salts), ion-pairing in which oppositely charged ions in solution can pair resulting in a net zero charge [65], diminished conductivity with increased solution concentration (the Debye-Hückel Theory of Electrolytes), incomplete salt dissolution due to salt-solution equilibrium for a specific salt species, and inadequate equilibration time for solubility of different salts (see [62] and their determination of EC stabilization time). Due to the complex interrelation of variables, conversion between results obtained using different methods is not recommended [66]. However, given the common practice of EC determination on a mass-based soil to water ratio dilution, and the calculation of threshold and yield estimates for agriculture based on the conductivity of the saturated extract (EC_e_), many studies have attempted to establish methods of measurement conversion (see Table 2 for equations and their published r^2^ values). These calculations have been variously successful, but should always be considered fallible with each estimate viewed as an approximate rather than precise value.

**Table 1:** Comparison of variations in electrical conductivity measurement.

Common Name	Common Abbreviation(s)	Soil to Water Ratio	Brief Description	Common Complicating Factors	Methodological influence on measured EC
Soil Water Extract	None	Variable	Water is extracted from the soil in the field by applying a vacuum to a soil surface. Soil water flows under pressure to a collection cup for measurement.	field moisture content, temperature	Ion concentration and resulting measured EC dependent upon field water content at the time of observation. Good for measuring temporal change in local EC, but not possible to compare measurements between overall locations. Conductivity increases 1.9% for every degree centigrade of increased temperature [61], and is often done at 25°C in laboratory settings. Many modern devices auto calibrate reported EC measurement based on a simultaneously measured temperature, but the specific device should be stated.
Extract of Saturated Paste	EC_e_; EC_se_; EC_SP_	Variable	Water is mixed with soil until it reaches saturation, and then this moisture is extracted under a vacuum from the solid soil. Amount of water used is dependent upon soil texture, and a general expectation is to recover 1/3 of added water upon extraction.	technician experience, saturation percentage variation, equilibration time, temperature	Method requires technician interpretation of qualitative attributes in sample production and is susceptible to variation based on experience. Insufficient equilibration time may result in an underestimation of EC.
Aqueous Mixture; Soil to Water Dilution	EC_1:1_	1:1	A given mass of soil is mixed with its equivalent volume of water. E.g. 10 grams of soil with 10 ml of water for a 1:1 ratio. It is then allowed to sit, often with occasional stirring, in order for the solution to equilibrate. Equilibration time, stirring method, and dilution ratio can be highly variable between studies.	equilibration time, extraction under vacuum versus direct measurement on soil to water mixture, dried & disaggregated sample versus ground sample, temperature	The concentration of ions in solution will decrease with higher dilution ratios and result in a lower measured EC. Direct measure of soil-water mixture will include conductivity due to clays whereas conductivity of an extract will be of ions alone. Insufficient of equilibration time may result in an underestimation of EC. This may be counteracted by grinding the sample, and thus increasing total surface area for dissolution, but may also increase hydrolysis of minerals and contribute to an artificial increase in EC.
	EC_1:2_	1:2			
	EC_1:2.5_	1:2.5			
	EC_1:5_	1:5			

**Table 2:** Conversion equations for estimating measured electrical conductivity on a saturated extract.

Original Measurement	Equation	Model r^2^	Specified Texture	Notes	Source
1:1	EC_e_ = 3(EC_1:1_)	--	--	Theoretical & likely to produce a higher predicted than necessary. Specific multiplication factors for conversion ranged between 2.78 and 1.6 based on the dominant salt type.	[64]
1:1	EC_e_ = 3.01(EC_1:1_) -0.06	0.98	Coarse	Suspension to Saturated Extract	[60]
1:1	EC_e_ = 3.01(EC_1:1_) -0.77	0.98	Medium	Suspension to Saturated Extract	[60]
1:1	EC_e_ = 2.66(EC_1:1_) -0.97	0.98	Fine	Suspension to Saturated Extract	[60]
1:1	EC_e_ = 1.85(EC_1:1_)	0.85	--	Only the regression without y-intercept was used in their validation. The regression equation of their validation set is also significantly different than that of their training set. Only their equation without a y-intercept is used here.	[67]
1:1	EC_e_ = 3.01(EC_1:1_) -0.06	--	Coarse	Specifically states they are not well calibrated & are a rough guide to interpretation only	[68]
1:1	EC_e_ = 3.01(EC_1:1_) -0.77	--	Medium	Specifically states they are not well calibrated & are a rough guide to interpretation only	[68]
1:1	EC_e_ = 2.96(EC_1:1_) -0.95	--	Fine	Specifically states they are not well calibrated & are a rough guide to interpretation only	[68]
1:1	EC_e_ = 1.93(EC_1:1_) -0.57	--	--	1:1 predictions were closer to EC_e_ than their other dilutions (1:2.5, 1:5)	[69]
1:1	EC_e_ = 2.72(EC_1:1_) -1.27	0.99	Sandy	--	[63]
1:1	EC_e_ = 2.42(EC_1:1_)	0.98	Sandy	--	[63]
1:1	EC_e_ = 2.15(EC_1:1_) -0.44	0.99	Loamy	--	[63]
1:1	EC_e_ = 2.06(EC_1:1_)	0.98	Loamy	--	[63]
1:1	EC_e_ = 2.23(EC_1:1_) -0.58	0.98	Combined	--	[63]
1:1	EC_e_ = 2.11(EC_1:1_)	0.98	Combined	--	[63]
1:1	EC_e_ = 3.35(EC_1:1_)	0.95	—	--	[69]
1:2	EC_e_ = 2.79(EC_1:2_) +0.71	0.91	Coarse	Extract to saturated extract	[60]
1:2	EC_e_ = 2.35(EC_1:2_) -0.36	0.95	Medium	Extract to saturated extract	[60]
1:2	EC_e_ = 2.16(EC_1:2_) +0.03	0.97	Fine	Extract to saturated extract	[60]
1:5	EC_e_ = 5.97(EC_1:5_) -1.17	--	--	1:1 predictions were closer to EC_e_ than their other dilutions (1:2.5, 1:5)	[69]
1:5	EC_e_ = 8.22(EC_1:5_) -0.33	0.98	Sandy	--	[63]
1:5	EC_e_ = 7.98(EC_1:5_)	0.98	Sandy	--	[63]
1:5	EC_e_ = 7.58(EC_1:5_) +0.06	0.99	Loamy	--	[63]
1:5	EC_e_ = 7.62(EC_1:5_)	0.99	Loamy	--	[63]
1:5	EC_e_ = 7.68(EC_1:5_) -0.16	0.98	Combined	--	[63]
1:5	EC_e_ = 7.57(EC_1:5_)	0.98	Combined	--	[63]
1:5	EC_e_ = 5.35(EC_1:5_)	0.96	Combined	See source for greater range of specialized conversions by texture & presence of gypsum	[70]
1:5	EC_e_ = 7.31(EC_1:5_)	0.91	none	--	[71]
1:5	EC_e_ = (EC_1:5_)(Q_1:5_/Q_e_)	--	sample	Q1:5 can be assessed as (500 + 6ADMC) for a 1:5 soil to water suspension (where ADMC is air dry moisture content expressed as kg/100 kg).	[61]
EC_e_	EC_1:1_ = 0.33(EC_e_) +0.06	--	Coarse	Specifically states they are not well calibrated & are a rough guide to interpretation only	[68]
EC_e_	EC_1:1_ = 0.33(EC_e_) +0.77	--	Medium	Specifically states they are not well calibrated & are a rough guide to interpretation only	[68]
EC_e_	EC_1:1_ = 0.375(EC_e_) +0.97	--	Fine	Specifically states they are not well calibrated & are a rough guide to interpretation only	[68]

## Review of salinity studies in Chaco Canyon

To evaluate existing studies of Chaco Canyon soils and their implications for agricultural productivity, we must first review the methods employed to determine soil salinity assessments. We particularly focus on those who have worked in the area of ‘downtown’ Chaco (the concentration of great houses in the area now encompassing the Chaco Culture National Historical Park), but also include work conducted ~30 km farther upstream at Pueblo Pintado as this analysis also falls within the Chaco Wash watershed and examines soils in the floodplain of the Wash. Research done in adjacent watersheds, such as at Kin Klizhin or the Escavada Wash, is not included as the emphasis here is the salinity of soils directly impacted by the Chaco Wash drainage regime.

### Main salinity studies

Benson [33,47] and colleagues [46] drew on EC measurements as a proxy for soil or water salinity on samples that were collected by those authors within Chaco Canyon. They argued that high salinity levels in the main floodplain of the canyon would create an osmotic potential so low that crops would wilt due to their inability to take up moisture. This argument has also been referenced by Benson and Berry [72] and Benson [32,73] (Note B in S1 Notes). Conductivities presented within these publications have been obtained in different ways:

1. Benson et al. [46] obtained values from a saturated extract (EC_e_), but the length of equilibration period, device used, and measurement temperature are not given.
2. Benson [33,47] used a non-extracted 1:1 soil to water ratio mixture allowed to equilibrate overnight, and states that an IQ Scientific Instruments pH/conductivity device is used, but does not indicate a specific model. Though a minor detail, the model determines whether the device auto calibrates conductivity based on temperature. Full conductivity results were presented in a supplementary table.
3. Salinity data discussed in Benson [73] were not collected or analyzed by its authors, but were drawn from the United States Department of Agriculture’s Natural Resources Conservation Service, which analyzed samples using EC_e_ (in accord to their own methods manual [59]).

In the first two cases, the measurements are used to construct Benson’s argument that soils of the Chaco Wash floodplain were too saline, while those of the side valleys could have permitted agriculture. Though the conclusion that soils in Chaco Canyon were unsuitable for agriculture is internally consistent across these publications, the data analyses and calculations suffer from internal inconsistencies and the questionable application of techniques.

#### Groundwater salinity is irrelevant to surface conditions

Benson et al. [46] used conductivity values for groundwater samples collected from wells within the Chaco Wash drainage to suggest that groundwater was a potential driver of salt accumulation in soils prior to the incision of Chaco Wash. These authors based their reasoning on the argument that the water table was within a meter or two of the surface in antiquity, and salts from the relatively high EC groundwater would have precipitated near the surface through evaporation from the capillary fringe, leading to soil salinization. Groundwater depth is significant because the zone directly above the water table, the capillary fringe, draws moisture upward against gravity due to the capillary attraction of water to soil pore linings and dry ped faces. If close to the ground surface, water can evaporate from the soil, creating an evaporative pump in which water continually moves upward to the capillary fringe from the water table, evaporates, and precipitates dissolved salts from that water into the soil [74]. However, Benson et al. [46] provided no evidence of a rise in the water table to within one or two meters of the ground surface.

It would seem that this argument for groundwater derived soil salinity is based on an unsupported assumption about the depth of the water table. Though it is not explicitly stated that the wells sampled for their groundwater EC data were within the modern incised wash, the coordinates provided fall within it. This conclusion is supported by Martin [75] where historic wells were noted as having been placed within the wash due to the inability to reach usable water supplies in deeper wells dug farther away from the wash, and he associated the remnants of wells still visible in the wash with those of the Hyde Expedition (1890s) and the National Geographic Expedition (1920s). Wells sampled in Benson et al. [46] had an average depth of just over nine meters below the wash surface, but ranged between seven and 16 m. Even if it is assumed that the level of the alluvial aquifer rose relative to the level of Chaco Wash, and that the wash during great house occupation was at the same level as the modern surrounding floodplain (4–5 meters higher than present), the water table would still be an average of 9 meters below the ground surface based on the depth to alluvial aquifer groundwater measurements in Benson et al. [46] and Martin [75]. Evidence suggests that a depth to groundwater of over 3 meters is enough to prevent evaporative losses [76]. Thus, even though groundwater in the alluvial aquifer does have high EC, there is no evidence that this water would have impacted soils near the surface regardless of wash incision. An eleventh century C.E. canal contemporary with Pueblo Bonito was two to three meters deep and would have intersected any water table at shallow depths, but no evidence from the recent reanalysis by Wills et al. [77] suggested continual water presence in the canal or bank collapse that would be likely if water were flowing out of the sediment into the canal. Furthermore, the focus on well water data in Benson et al. [46] ignores the fact that their *surface* water samples from Chaco Wash, a water source that, unlike groundwater, would have actually been used for agriculture, fall within salinity tolerance levels for irrigating maize agriculture.

#### Soil salinity measures, conversions, and comparisons

Ayers and Wescot [78], Ayers [79], and Maas and Hoffman [80] are cited variously by Benson et al. [46], Benson and Berry [72], and Benson [33,73] to support a salinity threshold of 1.7 dS/m (Notes C, D in S1 Notes), at which point maize productivity begins to decline. While Benson et al. [46] account for a decreasing yield beyond that threshold, the others present it in isolation, giving the impression of complete maize crop failure beyond that point, which is not the case (Note E in S1 Notes). There is no evaluation of the *progressive* nature of yield decline at EC values higher than 1.7 dS/m (see Table 3). For example, a value of 3.8 dS/m would result in a yield decline of only ca. 25%. Equally important, is a lack of discussion concerning the inherent issues of assessing agricultural production in past societies using declining percentages developed for yield maximization in modern agricultural conditions or crop species, even though we know that modern and prehistoric agricultural practices and cultivars differ considerably.

**Table 3:** This table shows estimated crop yield declines at particular soil or irrigation water conductivities. EC_e_ is a measurement on the extract from a saturated soil paste. EC_w_ is the conductivity of irrigation water with yield declines based on an estimated 15–20% leaching fraction. These data are always presented as guidelines, not definitive limits, and are for modern crop varieties. Given the range of tolerance within a given crop type, see squashes, it is possible that varieties used by Chacoan farmers were less susceptible than modern varieties largely grown in wetter climates. Data, except for sunflower, is from [81]. Amaranthus, found to be part of diets at Salmon Ruin and Antelope House, is considered a tolerant plant to salinity [82]. Chenopodium, Amaranthus, and Asteraceae were found to be significant diet contributions [82], and each is considered a halophytic, or salt adapted, plant.

	Yield Decline Percentage									
	0%		10%		25%		50%		100%	
Crop	EC_e_	EC_w_	EC_e_	EC_w_	EC_e_	EC_w_	EC_e_	EC_w_	EC_e_	EC_w_
Corn (*Zea Maize*)^1^ [81]	1.7	1.1	2.5	1.7	3.8	2.5	5.9	3.9	10	6.7
Sunflower (*Helianthus annuus L*.)^2^ [83]	4.8	/	6.8	/	9.8	/	14.8	/	24.8	/
Squash, scallop (*Cucurbita pepo melopepo*) [81]	3.2	2.1	3.8	2.6	4.8	3.2	6.3	4.2	9.4	6.3
Squash, zucchini (*Cucurbita pepo melopepo*) [81]	4.7	3.1	5.8	3.8	7.4	4.9	10	6.7	15	10
Bean (*Phaseo lus vulgaris*) [81]	1	0.7	1.5	1	2.3	1.5	3.6	2.4	6.3	4.2

^1^This is for sweet corn or grain corn. For forage corn, it is given as 1.8 with a decrease of 7.5% per 1 dS/m increase, not the 12% presented here.

^2^Based on the initial threshold and 5% seed yield reduction per unit dS/m increase.

However, the greatest problem with the discussion of EC in Benson [33,47] is the inappropriate conversions used to make the measured EC data comparable to the cited threshold values, which resulted in the calculation of erroneously high estimated EC values. The data in Benson [47] are conductivities measured on 1:1 soil to water ratio mixtures, but the values used to create the graphs, figures, and tables presented in the actual paper are listed in a separate “Cond S.P.” column (S.P. refers to Saturated Percentage, and thus “Cond. S.P.” is intended to be the equivalent of EC_e_). Cond. S.P. values were derived by multiplying the measured EC values by a factor of 1.85, but the paper does not mention the data conversion nor explain the rationale for this multiplication factor of 1.85 (Note F in S1 Notes). While the general relationship of lower EC in side-valleys relative to floodplain samples remains valid, the comparison to any outside threshold is questionable because of the decision not to reveal and explain the data conversion rationale and methods. Moreover, these unsubstantiated converted values are continually relied upon to suggest a meaningful detriment to the agricultural potential of the canyon by stating soil EC values are “non-optimal for the production of maize” without providing the necessary discussion of what “non-optimal production” in fact means [47].

In the response to Tankersley et al. [48] (which is described below), Benson [33] related thresholds for maize production to estimated conductivities experienced by plant roots at field capacity. He rightly reasoned that the amount of water during laboratory EC testing represents far more than a soil could hold against gravity and would result in a lower concentration of ions from dissolved salts in solution. Taking a sandy soil to be representative of all Chaco soils and estimating that such a soil would have a field capacity of 20%, Benson [33] multiplied the EC values in the paper by five to estimate soil water conductivity at field capacity. In the discussion of Chaco soil salinity, these multiplied values were then employed for comparison with threshold conductivities. Unfortunately, this approach includes several critical errors.

First, prior to any conversions applied to EC values in Benson [33] itself, the initial values, labeled ‘Cond S.P.’, were obtained by multiplying the 1:1 ratio by 1.85 in Benson [47]. As discussed earlier, this appears to have been done in an unstated attempt to convert laboratory measurements to comparable EC_e_ values. The exact source and conversions for EC data in Benson [33] were clouded by its referral of readers to Benson et al. [46] and Benson [47] for the salinity methodology. Benson [47] states that the EC values presented were obtained via a 1:1 ratio assessment, whereas conductivities in Benson et al. [46] were obtained on a saturated extract. Comparison of data used by the three publications shows that none of the EC data from [46] are presented or discussed in Benson [33] and citing [46] for methodology is misleading.

Second, Benson [33] multiplies his ‘Cond S.P.’ values by five in an attempt to estimate conductivities at field capacity. While this approach is theoretically appropriate given the paper’s assumption that field capacity is 20% of the water present when the soil is saturated, it fails to account for complicating factors involved in salt solution behavior, and compounds issues created by the multiplications done in the conversion steps mentioned above, resulting in exceedingly high EC values. In real world solutions, conductivity does not increase with ion concentration according to a theoretical linear relationship due to interactions among ions and their hydration shells. In a discussion of soil salinity assessed by aqueous electrical conductivity, Rhoads et al. [66] specifically state that the required conservation of mass assumption with changing water contents does not have *enough* validity to allow the EC at one water content to be determined “as the product of the EC at the second water content and the ratio of the two water contents”. This means one cannot be sure that the same number of ions remain in a soil solution after a reduction of water volume. Thus, the EC cannot be multiplied by the reduced fraction because, if the total number of ions is less than in the original solution, the actual EC of a soil solution after water reduction will be lower than the result of multiplication. In other words, one cannot take a measurement obtained at one moisture percentage, assume field capacity is 20% (i.e. the maximum amount of water a soil can hold against the pull of gravity 1/5 of its mass), decide that measured EC is one fifth of the desired conductivity, and then multiply the measured reading by 5 to get a field capacity EC measurement. Yet, this is exactly what is done in Benson [33]. The data errors created by this inappropriate process are further compounded by the fact that in Benson [47], 1:1 ratio values had already been multiplied by 1.85, meaning that Benson [33] presented conductivities whose original 1:1 EC values had been multiplied by a rather extraordinary and unjustified 9.25.

Third, and perhaps most critically, determinations of agricultural feasibility in Benson et al. [46] and Benson [33] are based on comparisons of highly concentrated salt solutions to threshold values determined from less concentrated solutions. In both papers, measured conductivities are converted to conductivity at either field capacity or wilting point, but then compared against a cited conductivity threshold for salinity whose value is from a saturated extract (EC_e_). Ayers and Wescot [78,81] and Ayers [79] provided collated soil conductivity measurements in EC_e_ values and water values as direct EC_w_, with the EC_e_ and EC_w_ values remaining constant across all publications. One of the sources used for these collations [80] was checked by the authors of this paper for method verification, and it specifically states conductivities were determined on the saturated extract of samples. These values were obtained by using test agricultural plots in which a studied crop was grown in soils of different salinities. Crop yield statistics are then compared against EC_e_ values obtained from soil samples in those test plots [64]. The justification in Benson et al. [46] and Benson [33] for attempting to determine EC at field capacity and wilting point is that the EC of soil water increases as the total amount of water in a soil decreases due to evapotranspiration. However, these same fluctuations in soil moisture occurred in the test fields that were used to establish the numerical relationship of salinity to yield declines. Though efforts were made to avoid accumulation of salts at specific depths in test fields, the irrigation schedule mimicked that used in agriculture [64]. Plants in these test plots would be exposed to fluctuating EC due to varying water content following an irrigation session and ensuing gradual drying. Thus, the resulting EC_e_ thresholds are directly comparable to EC_e_ from a location that would experience fluctuating water content. Given inclusion of that EC variability in the values presented by Ayers and Wescot [78], it is inappropriate to try to determine a conductivity value only at field capacity then to compare it to threshold values obtained from a saturation extract. Only if one were to convert Ayers’ EC_e_ threshold of 1.7 dS/m to a value at saturation percentage (e.g., if following the same approach as Benson [33], multiplying it by 5) would Benson’s [33] approach of multiplying conductivities even begin to be logically comparable.

In an earlier study, Benson et al. [46] assessed soil salinity by measuring conductivity on a saturated extract to produce EC_e_, values which would require no further alteration for them to be comparable to the paper’s cited yield thresholds. However, these EC_e_ values were converted in order to estimate EC at field capacity and wilting point, and those higher estimates were then compared against thresholds still at EC_e_. Since the actual soil salinity measurements for observed yield decreases were conducted on a saturated extract, and there is a significant decrease in accuracy associated with converting values from one concentration to another, it was neither appropriate nor necessary to try to calculate conductivity values at field capacity as is done in Benson et al. [46] and Benson [33]. The conclusions drawn by comparing the estimated conductivities in those papers against their cited threshold are unsubstantiated.

Benson et al. [46], Benson and Berry [72], and Benson [33,47] thus make critical errors in either data comparison or data calculation. In Benson et al. [46], this is reflected in a lack of clarity or adequate reasoning for the comparison among samples. In Benson [33,47], original measurements are used in ways that are not in accord with established salinity studies or practices. Also significant is the tacit presentation of salinity thresholds as a success-failure binary that ignores the reality that successful, though perhaps less productive, agriculture can be practiced in less than ideal soils. In total, the presentation of data is based on inappropriate methods of data comparison for electrical conductivity and inadequate recognition of the complexity of the interrelated factors involved in measuring soil salinity.

#### Other salinity studies in Chaco Canyon

Tankersley et al. [48] and Tankersley [53] presented both salinity data measured by electrical conductivity, as well as chemical assays on dry sediment to measure elemental composition. Using inductively coupled plasma optical emission spectrometry (ICP-OES) to determine the composition of local water, and x-ray diffractometry (XRD) on sediments (techniques not previously used in Chaco Canyon), they argued that the salts present in the soils are predominantly sulfates. Both papers concluded that most soils did not have salinities at concentrations high enough to significantly impact maize agriculture and that sulfates at the levels measured would, in fact, be beneficial. Their study, thus, starkly contradicts the work of Benson et al. [46] and Benson [47], going so far as suggesting favorable conditions for agricultural productivity based on Chaco soil salinity and chemistry.

However, there are also issues with the presentation of data in Tankersley et al. [48], making it impossible for readers to critically evaluate all of their claims. The first problem is the reporting of parts per million (ppm) for salinity results as converted by the Extech EC400: ExStik II instrument [84], which takes a conductivity reading and then applies a ratio to produce the value in ppm that the device actually displays for its total dissolved solids and salinity settings. Tankersley et al. [48] state that salinity data in the publication’s tables and discussion are determined from the device’s total dissolved solids (ppm) and conductivity (dS/m) modes. However, data presented in the paper are not the total dissolved solids values from the device, a setting which does allow a user-specified ratio, but are, in fact, from the device’s salinity setting, which does not. At issue is that the ratio applied in the salinity mode for this specific device is chosen per measurement by the device without user control, and can vary between 0.4 and 0.6. It is impossible for anyone with the published ppm data to determine the original 1:5 conductivity measurement because the ratio applied for conversion cannot be known (Note G in S1 Notes).

The second problem is that conductivity measurements were not done on bulk soil samples, but rather on 5 grams of the sand-sized (212–849 μm) fractions after dry sieving bulk samples [84]. Emphasis was on smaller fractions in that range, with a sequentially larger size fraction used if the smaller one did not contain enough material for analysis [84]. Use of a specific sieved size fraction instead of a bulk sample results in conductivities whose accuracy is uncertain. Because authigenic crystallization—the in-situ formation of new minerals—in a pedogenic context starts with very small size fractions, it is possible that analysis of sand-size fractions preferentially excluded precipitated salts, resulting in an artificially low conductivity. Given the non-standard method for their soil salinity assessment and the omission of the soil to water ratio, the conversion ratio of conductivity to ppm, and the device used, readers are unable to evaluate the salinity measurements presented in Tankersley et al. [48] in comparison to other soil research in Chaco Canyon or soil salinity studies in general.

In a recent paper [53], a new approach to studying soil salinity in Chaco Canyon is introduced through the use of energy dispersive X-ray fluorescence spectrometry (ED-XRF) on powder pressed sediment samples. This technique, like ICP-OES, has the benefit of providing highly precise elemental compositions for tested samples and presents a potentially revolutionary direction for soil salinity analyses seeking to identify the contribution or concentration of specific salt species. Currently, the greatest limitations are a lack of comparative material for contextualizing results from such techniques. Tankersley [53] found that average Na content for natural alluvium was 0.75%–1.65% and ranged between below detection limit and 1.66% for sampled deposits from canal sediments (operations C-01 and C-03). This is presented as evidence that Chaco Canyon soils have very low salt content, contradicting Benson [33].

While the averages presented in Tankersley [53] are numerically low, they cannot be related to potential agricultural productivity as claimed. First, ED-XRF measures the total elemental concentration of the specified element regardless of component minerals. While the analysis of Na was done with a focus on NaCl, a common, very soluble salt, there is no way to know from the presented data if the percentages of Na are from NaCl or other Na bearing minerals such as albite (NaAlSi_3_O_8_), a non-water soluble feldspar unrelated to soil salinity. Second, there is neither an established method of relating total sediment elemental compositional data to agricultural impacts (even if it could be assumed that all Na measured was part of the mineral salt NaCl) nor are there conversion methods for total sediment ppm data to relative EC_e_. Rough approximations for converting ppm data to EC values, such as multiplying an EC value by 640 to estimate salt ppm [85], would be based on the measurement of ppm data on a saturated extract. However, it is not possible to take the total sediment percent for an element such as Na and divide by 640 to estimate a rough EC_e_ because of the inherent differences between the mediums being analyzed (total bulk sediment versus a liquid water soil solution extract containing dissolved ions) (Note H in S1 Notes). Currently, sediments tested in Tankersley [53] cannot be definitively classified as either saline or non-saline and, ultimately, cannot be related to the existing salinity debate regarding agricultural production in Chaco Canyon.

Farther upstream on Chaco Wash from the Chaco Culture National Historical Park, Worman and Mattson [86] studied soil salinity near the Ancestral Puebloan great house of Pueblo Pintado. They presented EC measurements conducted on a 1:2 soil to water ratio solution extracted from 10 g of bulk sediment from samples taken along a single profile exposed along the modern wash, as well as comparative samples from the active Chaco Wash and the surrounding floodplain. Their results indicate salinity levels high enough to impact agricultural productivity in comparison to their referenced threshold, but they argued that, given the necessity of food production, an ancient population would have likely continued to engage in agriculture even if their yields were suboptimal. Conductivities from Worman and Mattson's [86] Profile 1, converted to EC_e_, are high enough to decrease yields between 50% and 100% according to the thresholds of Ayers and Wescott [78,81]. It is noteworthy that all of their samples had such high salinities, including their local reference samples, in comparison to those further downstream. This may be due to greater water availability in Chaco Canyon downstream from Pueblo Pintado because of the confluence of the Chaco Wash with two major secondary drainages, Gallo and Fajada Washes, as well as many minor tributaries.

### Agricultural feasibility in Chaco Canyon reconsidered

The floor of Chaco Canyon is known to be a geomorphically dynamic setting involving periods of incision and aggradation [87–91]. Existing salinity studies have largely involved samples collected independently of dated geomorphic units or archaeological material. Thus, it is possible that some soil profiles that extend to a meter or more in depth represent post-Ancestral Puebloan accumulation [92]. Higher salt levels may have accumulated in the past century and a half with the incision of Chaco Wash ([92,93] p437 of the latter). Salt accumulation or removal in soil can occur in a fairly short time span. For example, a study that examined an irrigated area in Turkey between 1966 and 2008 found an average decrease in conductivity of 2–5 dS/m at varying depths with maximum observed values falling from 20–22 dS/m to 7 dS/m [94]. Salinity studies of undated sediment deposition or an unknown pedogenic window—which is the case for all published salinity studies of Chaco Canyon to date—should be considered only in the broadest of terms in an overall discussion of local agricultural potential.

Though they are the largest sources of information for soil and water quality in the country, the descriptions for soils assigned to Chaco Canyon in the United States Department of Agriculture’s (USDA) soil survey of San Juan County [95] are not based on any sample data from the canyon itself [96], and there are only limited water quality records from the United States Geological Survey for Chaco Wash. Benson [47] produced a map of soil salinities at the surface and 1-m depth using a simple spatial interpolation on data obtained from the USDA but did not acknowledge that spatial variability in soil salinity is high in any setting or that soil salinity is heavily influenced by local topography and drainage. Divesting salinity data of their associated topographic and textural context makes spatial interpolation effectively meaningless. To assess potential salinity issues in Chaco Canyon soils, one must consult the soil series characteristics of this specific region (and this should only be used to form a first impression for an area’s soils). No soils have been described within Chaco Canyon itself; the geographically closest three soil pedons classified by the USDA were 55, 69, and 96 km from the canyon (see Fig 1). The closest of the pedons had high salinity values that influenced the visual effect of the interpolated maps in Benson [47]. Notable, this high salinity pedon was at the base of the Chuska slope, an area Benson [33] posits was a regional “bread basket.” According to the USDA, Chaco valley soils are identified as the Blancot-Notal (BT) association, with the Blancot series (55% of the canyon) described as nonsaline to slightly saline (0–4 dS/m) and the Notal series (25% of the canyon) as slightly to moderately saline based on depth (0–8 dS/m; [96]) (Note I in S1 Notes). The remaining 20% of Chacoan soils are described as a complex mix of slightly to moderately saline soils from additional series. Crop yields are not estimated for either the Blancot or the Notal series, but the Shiprock series—which has similar textural composition and identical calcium carbonate, gypsum, salinity (as measured by EC_e_), and sodium absorption ratios—is estimated to yield 150 bu of maize per acre under a high level of management (irrigation, fertilization, and other approaches considered as standard for USDA yield estimates) [95,97]. Thus, on the basis of soil composition estimates, Chaco Canyon has the potential to be quite productive if agriculture is approached in a managed fashion.

**Fig 2:**
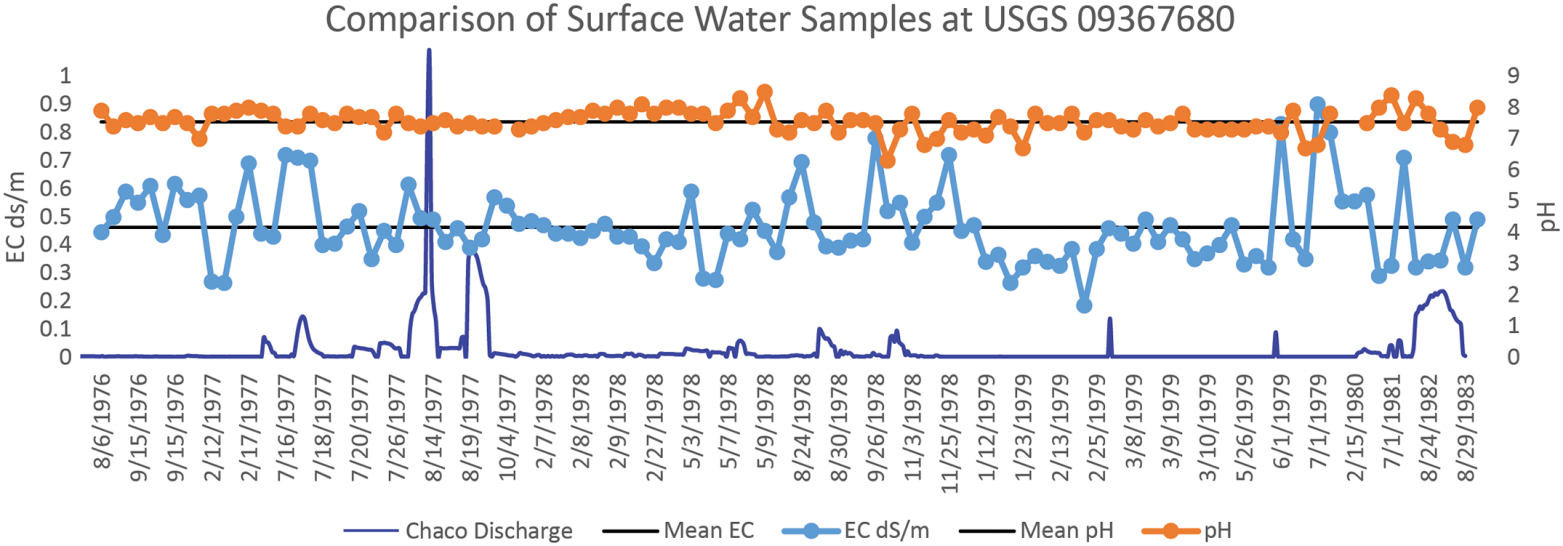
Shows the relation of key water characteristics for 113 observations between 8/6/1976 and 10/6/1983. The average for pH (7.55) and EC (0.46) are each indicated by a solid line behind each data type. Chaco discharge is shown for visual comparison of covariation between periods of increased flow and EC.

If used for irrigation purposes, the salinity of water is important: as the applied water is withdrawn by evapotranspiration, dissolved salts will precipitate out into the soil which can, over time, increase overall soil salinity. A USGS water sampling station (USGS 09367680) is located just downstream of the Chaco and Fajada Wash confluence. Though water quality data are only available from 1976 to 1983, there are samples from all months and seasons within this timespan. Data clearly show that the electrical conductivity of Chaco Wash water has an average of 0.46 dS/m, and its waters remain well below 1.1 dS/m (Fig 2) regardless of season or discharge. The composition of modern Chaco Wash water thus is not an impediment to agricultural production if used for irrigation. The leaching fraction (LF)—the amount of water applied in excess of evapotranspiration that is used to wash salts to depths below the rooting zone—could be as low as 0.1 or even 0.05, and the soil conductivity using Chaco water for irrigation would equalize at 0.97 dS/m or 1.47 dS/m respectively (using equation EC_w_ x 3.2 = EC_e_ for a leaching fraction of 0.05, and EC_w_ x 2.1 = EC_e_ for a leaching fraction 0.10, where EC_w_ is the conductivity of the applied irrigation water [81]). Put differently, 90 to 95% of the irrigation water from Chaco Wash, if applied to a field, could evaporate or be taken up by a plant and transpired, and the salt concentration in the soil would not increase year-on-year to a point that would have any negative impact on maize crop yields. These data show that the salinities of Chaco Wash waters are well within acceptable limits for irrigation agriculture.

Within the same timespan as the wash EC data, there are 42 laboratory measurements available for water composition. With the exception of a single reading, the sodium absorption ratio (SAR)—a measure of the amount of sodium relative to selected other dissolved solids—has a mean of 5.7, which is below the suggested maximum value for water used in irrigation (a value detrimental for clayey soils; coarser textures allow even higher values). Importantly, the SAR is typically below the level at which sodicity—the amount of sodium held in the soil—is a concern during the wet period critical for maize growth, from July to September (discussed in more detail below) (Note J in S1 Notes). Decreases in the ratio of sodium to sulfate (mg/L) correspond to this period, suggesting water contributed from surface runoff has a greater amount of dissolved sulfate than during drier periods when water in the wash may derive largely from groundwater seepage.

**Fig 3:**
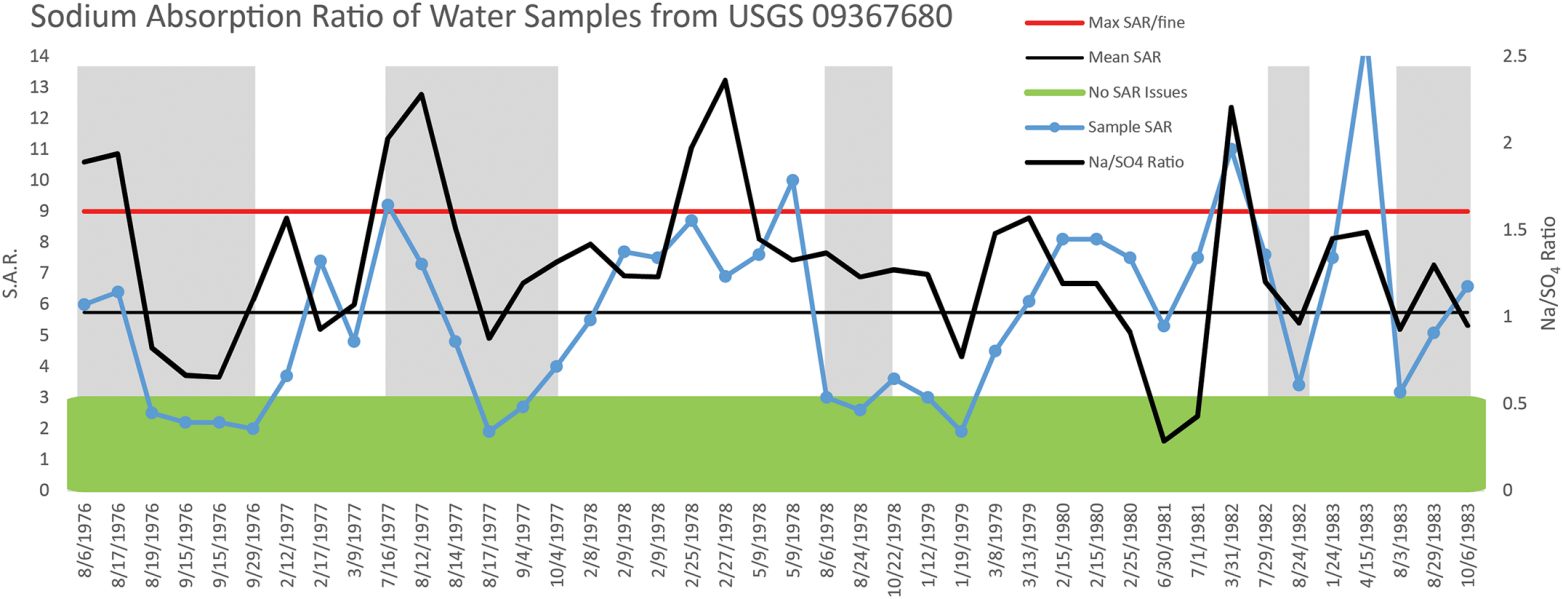
Shows SAR variation from 41 measurements from 8/6/1976 to 10/6/1983. Max SAR/fine—the highest flat horizontal line—is the maximum SAR value usable for irrigation on fine textured soils under any management practice and is the highest flat horizontal line. Mean SAR represents the value of 5.74. No SAR issues—the horizontal shaded area at the base—indicates that below a value of 3 there is no projected impact from the Na composition. The Na/SO4 Ratio is the simple ratio of the USGS data for each reported in mg/L. Shaded vertical bars indicate the seasonal period of precipitation at Chaco Canyon: July through October.

While the data initially suggest that the average salt composition of water in the Chaco Wash is mainly sodium due to a SAR greater than 1 for most months of the year, this does not take into account the highly seasonal flow of the wash. Given that most wash discharge occurs only during the rainy period from July to October and that it may be reduced to a dry bed or slowly evaporating pools, the majority of the actual water that flows in Chaco Wash has a *near* even ratio of sodium to sulfates and, at times, even has a greater sulfate component (Figs 2 and 3). The wash water sample presented in Tankersley et al. [48] has slightly higher sulfate than sodium values in accord with the sample being collected between late July to October (note in Fig 3 the Na/SO_4_ ratio often dips below 1 indicating sulfate enrichment). A lower SAR could be important if ancient farmers in Chaco Canyon displayed the same preference as the present-day Hopi for fields composed of a layer of sand overlying a finer textured subsoil (see [98]; [99] for field description and diagram). The use of irrigation water with a low SAR would prevent the formation of a silt cap at the soil surface that could increase the surface residence time of water and thus its loss due to evaporation. It would also avoid the collapse of ped structure in the lower, finer-textured horizon with subsequent perching of infiltrating water and the associated dangers of root rot or the rapid accumulation of salts at rooting depth.

Ultimately, maize yield is not uniformly susceptible to water stress across the entire duration of its growth. According to the Food and Agriculture Organization of the United Nations [100], maize is most susceptible to yield reduction due to water stress during the flowering and early cob formation periods and least susceptible during its ripening and vegetative periods. Bradfield [95] described Hopi practices of planting over an almost two month period between mid-April to mid-June in the Oraibi valley of Arizona ([101] p14, and [102] p236 also observed Hopi people began planting in April), a practice which takes advantage of higher soil moisture due to winter snow melt and protects against undue influence of either a late or early frost. Based on the National Oceanic & Atmospheric Administration’s daily climate normal report for station GHCND:USC00291647 at Chaco Canyon National Monument, if crops were planted at Chaco during that time window, they would capitalize on the two highest months of precipitation, July and August, during the crucially important flowering and yield formation periods.

Rather than prioritize data from any one specific study, the following discussion is based upon a compilation of all available soil salinity data for Chaco Canyon, including the unpublished conductivity (dS/m) data used in Tankersley et al. [48] on material collected in 2014 by the University of Cincinnati Chaco research project led by Vernon L. Scarborough. Conductivities are converted to dS/m where appropriate, and relevant conversions from Table 2 were applied to estimate EC_e_. Conversion methods that routinely estimated values below 0 are excluded on a per source basis. For example, the equation for converting 1:1 ratio values on medium textured soils in Franzen [68] produced many negative values, so while estimated EC_e_ using that equation are shown in S1 Table, they are not considered for interpretation. We also excluded the Zhang et al. [63] equation with intercept because a reading of 0 would still produce an estimated EC_e_ of 1.46, and we consider it to be an unrealistic inflation in comparison with all other intercept values. Here the average of all accepted estimates is used for discussion rather than favoring any particular method. Results for each individual conversion can be found in S1 Table. Crop yield estimations, using data provided by Katerji et al. [103], show a <1% decrease in agricultural yield for soil conductivities between 0.8 and 1.8, a 21% decrease for a loam at EC_e_ 3.0, an 11% decrease for a clay with EC_e_ between 0.8 and 1.9, and a 24% decrease for EC_e_ 3.7, which are all very similar to estimates presented in the literature [78,79,81]. Given such consistency across sources and recent supporting results, we use the threshold values presented in Table 3 to consider the extent to which salinity may have reduced agricultural yields in Chaco Canyon.

Though 66 locations have been sampled for soil salinity in the general location of ‘downtown’ Chaco, only 23 are within the Chaco Wash floodplain. Of those 23, 14 comprise distinct areas that can be considered to sample the wider spatial variation in soil salinity across the entire Chaco Wash floodplain, which is the area Benson [33] believed to be entirely unsuitable for maize agriculture. The other samples were taken from within tributary rincons or on top of the mesas. A “distinct area” is considered here to be a sampled location in which there are no other samples within 100 m. There are several sample groupings with locations within 100 m, and, though clustering of sample locations illustrates local variability in soil salinity, it does not provide a complete picture of regional soil salinity. This distinction is made to highlight the relatively small spatial coverage of sampling within the entire Chaco Wash floodplain. The 14 distinct areas are evenly divided between locations with analyses at multiple-depths and single-depth samples. The latter are less useful since they provide less information about location variability. Chaco Wash floodplain and rincon canyon floor sediments exhibit great spatial variation in soil salinity; variability is also present within some individual depth profiles. Several samples collected at multiple depths from the floodplain indicate little to no yield decrease, while locations nearby may exhibit salinity levels capable of reducing maize yields between 10 and 50%. Four locations—two single depth and two multiple depth profiles—have conductivities high enough to effectively prohibit agricultural production (see S1 Table for all data).

**Fig 4:**
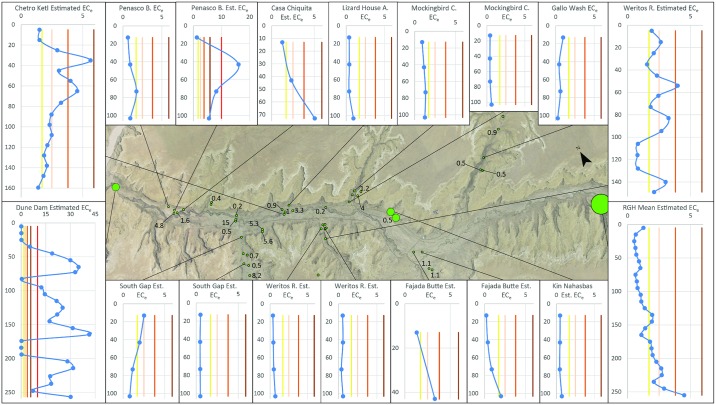
Figure shows the location for all known soil salinity samples in the main are of Chaco Canyon. Larger circles are to avoid providing precise location information for non-public archaeological areas. Selected profiles are presented with values for single depth samples shown next to their location. Blue circles, connected by a simple smoothed line for visual interpretation, represent estimated EC_e_ values in profiles. For sources that specify a depth range for specific samples, point depth is the range midpoint. For each salinity graph, the Y-Axis is Depth (cm), and the X-Axis is Estimated EC_e_. Vertical lines represent varying yield decrease thresholds for maize (moving left to right): Yellow = 0%, Peach = 10%, Orange = 25%, Brown = 50%, and (when shown) Red = 100%. See S1 Table for raw and converted data.

Soils within Chaco Canyon, NM display similar patterns of spatial variations in salinity to those identified in modern agricultural fields in other arid regions. For example, a study of soil characteristics for an area under cultivation bordering a roughly 10 km stretch of the Givy Chay river in Iran showed conductivities ranging from below 1 to 14 [104] (Note K in S1 Notes). Sampling of an agricultural field in Turkey (whose irrigation water has a conductivity of 0.980 dS/m, twice the Chaco Wash average) showed a soil conductivity variation of up to 2 dS/m across a distance of only 40 m [105]. Both areas in these studies were used to grow a mixture of maize and other crops. At a modern depth of 40 cm, soils in Chaco Canyon can range from 0.94 dS/m to 16 dS/m over a distance of 225 m (see Peñasco Blanco samples in Fig 4). While there are a few locations or depths at which the modern measured conductivity is high enough to completely prohibit food crop yields, these are not spatially continuous. Crops have also been shown to increase water usage from soil depths with lower salinity in situations when certain depths have salt concentrations high enough to impact plant usage of water [80].

Such variability is especially important given (a) previous treatment of Chaco agriculture as an all or nothing practice and (b) ethnographic accounts that document that Pueblo farmers employed a variety of agricultural strategies in an attempt to account for production variation and ensure a successful harvest. Thus, before concluding that almost all Chaco maize came from the Chuskas, as does Benson, one must ask several difficult to answer, but relevant questions. Was an Ancestral Puebloan farmer more likely to grow maize in Chaco Canyon even if yields on *some* fields were decreased 10% to 50% or rely on fields sown 70–80 km away requiring harvests be transported back to the canyon? Would they have chosen not to engage in agriculture because some areas were good for maize while other fields may only be productive for other crops, such as squash or sunflower? Hopi farmers are known to engage in a variety of field strategies, including dune fields and irrigated plots, to take advantage of such different opportunities [99]. They also plant multiple cornfields in a variety of locations, fully expecting that some will fail to produce well and anticipating year-to-year yield variability [101]. For some rain fed fields, crops could fully fail to mature 25–33% of the time [98,106]. Even in our modern market system, there is a wide variation in average field productivity. For example in 2015, South Carolina had an average per acre corn yield 45% lower than the US overall and 50–55% lower than the most productive states [107], yet local farmers persisted in growing corn. Such disparity in relative productivity found in a market-based, globally integrated agro-economic system designed for profit, not survival, highlights the dangers in treating agricultural feasibility as an all or nothing binary. Risk minimization strategies documented in Puebloan agriculture suggest that ancient farmers would have anticipated variable agricultural productivity across space and would have taken steps to account for less productive fields. In the next section, we discuss other limitations to the large-scale importation of maize to Chaco Canyon.

### Implications for Chaco population, social organization, and Chuska connections

Much of our understanding of the residents of Chaco Canyon can be traced to the unique history of archaeological research. The most extensive excavations of great houses occurred in the 1890s and 1920s and focused on the near complete excavation of Pueblo Bonito, the largest great house [108, 109]. Although National Park Service projects and field schools based at the University of New Mexico operated between 1940 and 1970, the next major fieldwork—the Chaco Project—occurred in the 1970s and focused on survey, excavations of many small sites, and testing of the great house of Pueblo Alto (e.g. [3,29]). Analyses of the materials from Pueblo Alto led to the introduction of the pilgrimage model [5]. Ultimately, however, the excavations by the Chaco Project exposed few great house rooms, although descriptions of the results of this fieldwork were extensively published. Recent research has largely focused on the Pueblo Bonito excavations through the detailed examination of archival records (chacoarchive.org), reanalysis of collected materials, and re-excavation of specific contexts (e.g. [1,4,7,11,110]).

While the idea that Chaco Canyon residents required provisioning from outside areas to survive is not new (e.g. [5,36,111–115]), it continues to be recast to explain the development of a complex sociopolitical system within this seemingly marginal environmental setting. Based on the low productivity postulated for maize [32,46,47,72,73] and documented material connections to the Chuska Mountains [11,35,116–121], Benson [33] claimed that the Chuskas served as a “bread basket” for Chaco Canyon residents (see also [32,34,46,72,122]). Benson’s [33] argument is based on two main lines of evidence: the sourcing of corncobs found within great houses and high soil salinity measures implying limited agricultural potential on the Chaco Wash floodplain. While we do not doubt that some food was brought into Chaco Canyon, the source and intensity of that importation remains controversial and claims of extensive provisioning from the Chuska Mountains, in particular, deserve further scrutiny.

Researchers have conducted strontium isotope analyses on corncobs from several locations within Chaco Canyon, including four great houses [32,46,72,122]. These studies identified multiple samples that could possibly source to areas of the Chuska Mountains. Specifically, five corncobs predating 1130 CE—the main occupation period—have been tested from Pueblo Bonito and sourced to either side valleys within Chaco Canyon, areas along the Chaco River, or the Chuska Mountain slopes. Tested cobs dating to post 1130 CE have been variously sourced to either the Tohatchi area of the Chuska Mountains, the Totah, Lobo Mesa, or elsewhere ([34] p186). Several important factors, however, need to be considered in interpreting and extrapolating from these results (see also [39] pp139-140).

First, the analyzed samples represent a very small proportion of the maize consumed within Chaco Canyon—the five corncobs dating to the main occupation of the canyon represent no more than a small fraction of 1% of the corn consumed by one individual in a single year—and should not be taken as sufficient evidence for the degree of importation suggested by Benson [32–33]. Additionally, these pre-1130 CE corncobs were all found within Pueblo Bonito. The material assemblage from Pueblo Bonito includes unprecedented quantities of non-local materials such as turquoise, shell, and macaws that have not been recovered from other sites within the canyon ([123] p.127, [124] p.85-86). The location and preservation of unburned corncobs within Pueblo Bonito may indicate that they were used ceremonially, which could preferentially favor the selection of nonlocal materials [125,126]. One should, thus, be very cautious about generalizing from Pueblo Bonito to other Chaco Canyon settlements, particularly the small house sites that vastly outnumber great houses and may have been the residences of most of the inhabitants of Chaco Canyon. Second, as noted by Grimstead et al. ([34] p185), strontium isotope analyses can exclude source areas, but it is difficult to determine a precise source location. Statistical analyses performed by Drake et al. [127] indicated that maize from within Chaco Canyon cannot confidently be sourced to any single region in the greater San Juan Basin due to substantial overlap in strontium signatures among regions. Specifically, Chaco Canyon and the Chuska Mountain region are not readily distinguishable. Thus, the pre-1130 CE corncobs could have been grown in side valleys of Chaco Canyon or nearby locations along the Chaco River, a conclusion that was favored by a member of the original study [125]. Finally, most of the post-1130 CE samples are irrelevant to discussions of the construction and occupation of the great houses as they largely represent later reuse of the pueblos.

Additionally, analyses of oxygen isotopes were used to suggest long-distance importation of both small and large mammals from higher elevations [35]. More recently, Hamilton and colleagues [128] found no difference between archaeological and modern small faunal samples that were collected within Chaco, although large mammals still appear to have been sourced from higher elevations. Given the noted ties of Chaco Canyon to the Chuska Mountains and other surrounding regions, the import of some maize and large mammals for ritual events or social gatherings was probable [53,129,130]. Social networks throughout the Southwest were crucial means for coping with environmental variability and the inherent risks associated with agriculture in this arid environment (e.g. [131]). As such, relationships with other regions were likely imperative for Chaco Canyon residents; however, these relationships were unlikely to override the need for local food production, and there is no clear evidence to suggest that the Chuska Mountains, rather than closer productive areas within the Chaco River drainage basin, served as the main source of imported maize.

Researchers have long debated the efficiency and likelihood of long distance material transport in the archaeological record [132–135]. Lightfoot [134] found that transporting staple foods on foot over 50 km is no longer efficient for society. In particular, the caloric needs of porters are a limiting factor [136], as personal consumption would deplete 0.5–1 kg of food daily ([137] p4). While these results have been heavily critiqued (e.g. [132,135]), ethnographic cases of long distance transport generally concern the movement of goods or specialized foods, such as salt or meat (e.g. [124,138–141]), an exception acknowledged by Lightfoot ([134] p335). Alternately, Drennan’s [132,133] work suggested that the transport of staple foods was possible between more distant locales, but noted, “ordinarily we should expect transport of such staples to be restricted to substantially shorter distances” ([132] p110). This statement seems particularly important given that accounts of long-distance transport of staple goods in Prehispanic North America are from Mesoamerican societies and time periods when there was marked occupational specialization that almost certainly would have impacted the distances over which goods could be transported.

Understanding the social implications of transporting a staple food requires a more detailed assessment of the local demographics and social conditions. To further unpack the implications of large-scale maize importation from the Chuskas, we present a number of estimates for the labor investment necessary to move the required amount of maize to Chaco Canyon (Tables 4, 5 and 6). Calculations reflect two frequently used estimates for canyon population totals: 2,000 and 5,500 [33]. Researchers commonly conclude maize accounted for between 70% and 90% of the diet in the Pueblo Southwest around this time, although certain high-status individuals may have consumed higher proportions of protein [43–45]. Based on maize comprising 80% of the diet, our estimates assume individual consumption of 169 kg annually, as determined by Matson [42] and reported in Benson [33]. Furthermore, Benson [33] calculated 209 people could reasonably be sustained by Chaco agriculture. To account for the local production of maize estimated by Benson [33], we removed the amount required to feed 200 individuals (33,800 kg) from our calculations of the annual maize needed to support the estimated population numbers. Neither the weight of containers that would have been necessary to transport maize nor the food consumed by individuals during these trips were included in these calculations. Our estimates thus reflect the minimum possible expenditures of time and labor.

**Table 4:** Estimated time and labor efforts for Chuska residents to transport maize to Chaco Canyon based on varying population sizes, carrying capacities, and travel times. Drennan [132] used loads of 20 and 50 kilograms in his studies of long distance transport. The final weight estimate derives from Malville [131], as reported in Windes and McKenna ([142] p136).

Weight Carried per Person per Trip (kg)	Chaco Population of 2,000: 304,200 kg Maize		Chaco Population of 5,000: 895,700 kg Maize	
	Percentage of 10,625 Chuska Population	Percentage of 17,000 Chuska Population	Percentage of 10,625 Chuska Population	Percentage of 17,000 Chuska Population
20	143%	89%	422%	264%
50	57%	36%	169%	105%
100	29%	18%	84%	53%

**Table 5:** Estimated time and labor efforts for Chaco residents to transport maize to the canyon based on varying population sizes, carrying capacities, and travel times. Round trip lengths are based on a distance of 85 km between Chaco Canyon and the Chuska Mountains. Six-day trips were suggested by Benson [33] based on traveling 26.67 km/day, while 9.5 and 27 days—assuming a speed of 18 km/day and 6.34 km/day respectively—were derived from travel times determined by Malville [137] and reported by Windes and McKenna ([142] p136). Drennan [132] used loads of 20 and 50 kilograms in his studies of long distance transport, both of which are paired with Benson’s assertion that the trip could be made in six days. The second two weight estimates derive from Malville [131], as reported in Windes and McKenna ([142] p136) and are directly linked to the trip lengths determined in the first column. Number of trips and travel days are rounded based on the completion of an entire trip.

Round Trip to Chaco (days)	Weight Carried per Person per Trip (kg)	Chaco Canyon Population of 2,000: 304,200 kg of Imported Maize			Chaco Population of 5,000: 895,700 kg of Imported Maize		
		*Number of Trips*	Trips by 100% of Population	Trips by 25% of Population	*Number of Trips*	Trips by 100% of Population	Trips by 25% of Population
6	20	15,210	8 (48 days)	30 (180 days)	44,785	8 (48 days)	33 (198 days)
6	50	6,084	3 (18 days)	12 (72 days)	17,914	3 (18 days)	13 (78 days)
9.5	50	6,084	3 (29 days)	12 (114 days)	17,914	3 (29 days)	13 (124 days)
27	100	3,042	2 (54 days)	8 (217 days)	8,957	2 (54 days)	7 (189 days)

**Table 6:** Estimated transport time and labor efforts for transport of maize to the canyon by seasonal residents living 6 months in the Chuskas and 6 months in Chaco Canyon based on varying population sizes, carrying capacities, and travel times. In this case, the amount of required maize is halved to represent only seasonal occupation within the canyon. Round trip lengths are based on a distance of 85 km between Chaco Canyon and the Chuska Mountains. Six-day trips were suggested by Benson [33] based on traveling 26.67 km/day, while 9.5 and 27 days—assuming a speed of 18 km/day and 6.34 km/day respectively—were derived from travel times determined by Malville [137] and reported by Windes and McKenna ([142] p136). Drennan [132] used loads of 20 and 50 kilograms in his studies of long distance transport, both of which are paired with Benson’s assertion that the trip could be made in six days. The second two weight estimates derive from Malville [131], as reported in Windes and McKenna ([142] p136) and are directly linked to the trip lengths determined in the first column. Number of trips and travel days are rounded based on the completion of an entire trip.

Round Trip to Chaco (days)	Weight Carried per Person per Trip (kg)	Trips by 100% of Chaco’s Population to Import Maize for 6 Months
6	20	3.5 (21 days)
6	50	1.5 (9 days)
9.5	50	1.5 (14 days)
27	100	0.5 (14 days)

The importation of maize to Chaco Canyon could have been achieved following three distinct social scenarios. First, individuals living in the Chuskas could have brought maize into the canyon in order to support local residents. Assuming a minimum of 169 kg of maize was consumed by individuals annually [33,42], between 3,000 and 45,000 individuals would need to make trips to the Chuskas each year to transport enough maize to feed the local population. The pilgrimage model assumes that individuals visited the canyon for ritual events and brought supplies with them on these trips (e.g. [5,24,25,36,143]). To participate in social and ritual activities, at least a portion of the transported maize would have been necessary to feed the visitors throughout their stay, and additional trips might have been required to fully supply the canyon’s residents if pilgrims stayed in Chaco Canyon for more than a few days. Second, the Chaco residents themselves could have travelled to the Chuskas to collect their annual supply of maize. If residents from Chaco Canyon transported their own maize, the entire population would have needed to spend 18 to 54 days (2–8 trips) travelling annually (Table 5). Either scenario indicates that a substantial amount of labor and time would have been necessary to minimally support the canyon’s residents.

For comparison, Malville’s ([135] p232) research in Nepal, which she used to argue in support of the possibility of long distance transport of materials to Chaco Canyon, found that each household made a single annual long-distance trip to replenish goods. While our estimates could align with the Nepal criteria, they only fit expectations when we assume every individual would have been able to act as a porter, an unlikely scenario for children and elderly or sickly individuals. The majority of the porters studied by Malville were males hired by shopkeepers to transport goods [137], and Hagen [144] observed that only about 20–25% of the total population in Nepal made an annual trip to procure goods ([135] pp232-233). If 25% of the population from Chaco Canyon transported maize from the Chuska Mountains, between 72 and 217 days, or 7–33 trips, would have been necessary. Using the estimated population ranges for the Chuska Mountains presented by Benson ([33] p13), only one possible scenario, which assumes the minimum Chaco population, the maximum Chuska population, and the maximum amount of weight carried, would require less than 25% of the Chuska population (18%) to serve as porters. All other scenarios exceed that threshold. Of the 16 possible scenarios calculated, four require < 50% of the Chuska population and five require < 100%, while the remaining seven suggest that all individuals would need to make more than one trip to transport sufficient maize to the canyon (Table 5). As noted earlier, these calculations are underestimates as the weight of containers and the food consumed by porters during travel and/or participation in social events within the canyon are not included. While the extreme values produced by these calculations are unlikely to be accurate, it is reasonable to assume that most Chuska residents would have needed to make at least one to two trips annually to support the inhabitants of Chaco Canyon, suggesting a much higher investment than has been documented ethnographically in Nepal [135,144]. This extent of provisioning from the Chuskas would imply that Chaco Canyon was a powerful polity that could demand the importation of food, such as existed in Mesoamerica where longer distance transport of foods was more common [132,133]. While hierarchy likely existed (e.g. [1,21,145]), there is no reason to suspect the canyon was powerful enough to extract resources from surrounding regions to this extent.

A third possibility is that the occupation of pueblos within Chaco Canyon was seasonal and the residents of these sites lived and farmed on the Chuska slopes during the growing season [10], a pattern noted for historic Navajo farmers in the 1900s [146]. Assuming that individuals spent six months in each location, the maize required to feed the population while in Chaco Canyon would be cut in half. If the entire population adopted this residence pattern and each individual could carry the maximum of 100 kg, seasonal occupants could have transported the maize they required for the winter months back to the canyon in a single trip. Otherwise, one to three additional trips would be needed to return with enough food for the season (Table 6). While these numbers appear somewhat more reasonable than the other scenarios, they remain underestimates due to the unlikelihood that every individual within a population would have been physically able to transport food. Additionally, the extensive occupation of the Chuska slopes during the Pueblo II period makes substantial use of farmland by Chaco residents unlikely (e.g. [147,148]).

While possible, investment of the scale needed to transport maize annually by a large portion of the healthy adults living within Chaco Canyon or the Chuska Mountains would be unprecedented in the Southwest, despite the importance of long-distance trade for historic Pueblo groups [141]. The time and labor required to transport this quantity of material annually also has significant social implications that cannot be ignored. Food transport would greatly impact the labor available for the performance of other activities. Chaco populations invested significant time and effort on construction, craft production, and, although limited in this scenario, local agriculture [3,10,149]. For instance, many items, such as wood beams, found within Chaco Canyon were likely acquired, processed, and transported by Chaco residents themselves [52,142]. Reliance on distant populations for staple food supplies would create an extremely inflexible system. While social networks can aid in survival through poor agricultural years (e.g. [131]), a single bad maize yield in the Chuskas would have had the potential to devastate residents of both regions. Judge ([5] p243) himself noted that the pilgrimage system would have “become increasingly vulnerable to environmental fluctuations” as it developed.

Additionally, the intensity of travel required to move between 300,000 and nearly 900,000 kg of maize annually seems unlikely to occur with little to no material trace. While roads emanate from the canyon that could suggest regular travel and transport, their identification and function have been widely debated [150,151]. Many researchers have suggested that the roads served a largely economic purpose, tied to the redistribution or importation of foods (e.g. [152–154]), although others doubt their use for material transport (e.g. [155]). Alternatively, Wills and Dorshow ([39] p153, [152]) recently hypothesized that at least some road segments located near great houses could have served as water control features diverting and directing runoff (see also [156]). Annual trips to move maize at the scale necessary to supply the residents of Chaco Canyon would likely have resulted in significant evidence of that transport, but the density of artifacts found along road edges is low, and features, such as hearths, that may be associated with campsites have not been identified ([151] pp46-47, [157]). Furthermore, while multiple roads emanate west of the canyon, no confirmed road directly connects Chaco Canyon with the Chuska Mountains ([158] p213). Thus, there is no clear evidence to support the extensive transport of maize between these two regions.

Most archaeologists argue that Chaco Canyon served as a ritual center for the region, providing powerful symbolism, ideology, and social network ties [5,15,22,159]. In part, symbolic capital might explain the lack of evidence for materials being exported from the canyon in exchange for the vast quantity of imported materials (see [5,160] for a discussion of the exportation of finished turquoise ornaments). From such a perspective, residents of the Chuskas would have provided the labor and land to produce maize to support the canyon in exchange for access to and participation in ritual events and social gatherings. However, a large portion of the population within Chaco Canyon likely resided in small sites, similar to other pueblos during that time. Symbolic capital may have served as enough reason to support a small priestly population residing within the great houses (*sensu* [5,29,30]), but there is no obvious rationale for distant populations to also have provided the maize consumed annually by residents of the small sites. While the presence of certain prestige and nonlocal goods in Chaco is clearly tied to its social prominence, it is difficult to justify the provisioning of thousands of individuals with their staple food source annually for several centuries along those same lines.

In part due to doubts held by researchers such as Benson about the potential for successful agriculture, ancient agricultural systems in Chaco Canyon have received very little attention overall ([161] p35). An analysis of the titles of publications listed in the Bibliography maintained by the Chaco Research Archive [162] indicates only about 2% (64 of 3096) explicitly reference agricultural practices (Note L in S1 Notes). Despite limited attention to the topic, Chaco residents would likely have exploited a wide range of agricultural practices and systems to improve productivity of the area. Water, which is always a finite and valued resource in the Southwest, could have been effectively captured through the use of multiple styles of farming, including floodwater farming, dune fields, akchin fields, and irrigation systems ranging in form, such as check dams and canals (e.g. [56, 86, 98, 99, 101, 161]; see [161] for a more thorough discussion of agricultural practices within the canyon). The employment of diverse and localized farming strategies would have helped manage risk and ensure a decent crop was produced most years.

In a related study, Dorshow [163] modeled the agricultural suitability of a large portion of the Chaco Core and estimated a potential yield of 123,520 kg of maize annually, or enough to support about 730 individuals, from almost 5,000 ha of fields. This study did not encompass all of the Chaco Canyon great houses or likely field areas and applies conservative estimates of yield and plant spacing. Cross-cultural studies suggest agricultural fields could be maintained four to five kilometers away from a village, although fields farther than one kilometer show a decline in caloric return [164]. In the late 19^th^ century, Bradfield [98] documented 2,000 Hopi individuals consuming over 300 kg annually were fed from less than 1,000 ha, suggesting the arable area throughout Chaco would have been sufficient to largely support the local population. Local agricultural potential is further supported by the relative success of historically documented farming of the canyon by Navajo occupants (e.g. [87, 146], [108] pp53-62, [161] p42]). Additionally, although largely used to highlight the difficulty of local agricultural production, experimental plots by the Chaco Project in the 1970s demonstrated the relative success of maize planted on the floor of the main canyon [54].

While more detailed publication of excavation data on Chaco Canyon water management is needed, recent research, building upon the extensive documentation and excavation of water control features by Vivian (e.g. [13,55,165,166]), has furthered our understanding of the diverse water management strategies that were likely practiced in and around the canyon to enable farming through the exploitation of diverse and localized strategies [39,86,161,163]. Canals originally documented in the 1970s and located at the west end of the current National Park Service boundary were recently re-exposed to obtain direct dates through Optically Stimulated Luminescence [167]. These results confirm canal use from the 8^th^ through the 11^th^ centuries ([168]; see also [76] for a discussion of canals around Pueblo Bonito). Thus, new studies strongly support the use of varied agricultural practices in and around Chaco Canyon that would have increased the productivity of the area.

In summary, while the canyon was certainly never an ideal setting for maize cultivation (see [54]), Puebloan people, today and in the past, have creatively adapted to local conditions in order to minimize the risk of these environments and practice successful agriculture. Given the extreme transport costs and lack of evidence for intensive exploitation of Chuskan agricultural fields, we conclude it is unlikely that nonlocal maize composed the bulk of food consumed at Chaco. Reassessments of soil salinity numbers suggest dynamic and spatially variable conditions for maize agriculture within the canyon that could have been exploited effectively through the use of multiple types of fields and effective water management as has been documented within the canyon (e.g. [13,39,86,161]). Thus, a reconstruction of Chaco society that involves local maize cultivation supplemented annually to varying degrees by other regions seems a more appropriate fit for the archaeological data.

## Conclusions

Studies of potential past agricultural productivity are built on a number of assumptions and often rely on assessments of modern agricultural conditions to estimate past potential. These practices are certainly necessary and useful, but applications of modern quality standards must be treated carefully when applied to archaeological contexts. Care must also be taken when using modern geochemical signatures as a gauge for those of the past, especially when analyzing a characteristic such as soil salinity, which can be rapidly altered by changing environmental conditions. For example, Benson ([47] p96) states that “agricultural productivity and field-life calculations should be considered only in an illustrative sense, given their assumptions.” Caution seems particularly justified when results of agricultural productivity studies suggest extreme limitations that defy reasonable expectations.

Though electrical conductivity (EC) is an attractive technique because of its relative laboratory simplicity and low cost, not accounting for the specifics of the applied methodology, such as careful selection of tested material, soil to water dilution ratio, equilibration time, conversion equations for inter-study comparison, and instrumentation, can create misleading results. We recommend that researchers either use only established approaches to allow for better data comparability or specifically demonstrate that their approach does not produce results that vary from established methods. If possible, it is also highly recommended that the actual types of salt present in the solution after measurement be determined to better interpret the result. We have concluded that failure to follow established procedures has led to unverifiable, misleading, or even incorrect conclusions regarding soil salinity in the discussion of agriculture in Chaco Canyon.

Any attempt to utilize soil salinity as a definitive assessment of agricultural productivity should first be based on a far more rigorous spatial sampling program with greater concern for temporal control than has been conducted to date at Chaco Canyon. Based on a small number of undated samples from the Chaco Wash floodplain, Benson [33] concluded that the entire area was overly salinated, ignoring both the high spatial variability of soil salinity and the lack of a clear association between the samples and the Ancestral Puebloan occupation of the canyon. Widely dispersed, undated individual samples should not be extrapolated to form broad assessments of potential agricultural productivity throughout history. Instead, extensive sampling of areas of interest should be undertaken in combination with dating techniques to provide temporal control prior to the determination of past agricultural potential.

Most soil salinity studies of Chaco Canyon have fed into, or been reinforced by, long held beliefs about the marginality of the environmental setting in order to suggest intensive maize agriculture was impossible. In some instances, the reasoning has come dangerously close to being circular; estimates for low agricultural potential have in turn encouraged low population estimates, while supporting arguments for the canyon’s reliance on neighboring areas for food with little consideration of the social implications of such a system. For instance, the intensive transport schedule that would have been necessary to supply maize to the canyon’s residents would have created an inflexible and delicate arrangement that would have been vulnerable to any, even short-term, changes in environmental or social conditions.

Upon careful reassessment of all available salinity data for Chaco Canyon in relation to known agricultural yield gradients for salinated soils, we disagree with many previous studies suggesting that these soils are largely unsuitable for agriculture. Our reevaluation of salinity shows that canyon soils display the same spatial variability identified in other studies of modern, agriculturally productive fields. Even in many of the areas where salinity is high enough to have been a deterrent to maize potential, the salinity levels are still within the range tolerated by other foodstuffs grown and consumed by Ancestral Puebloan peoples, such as squash and sunflower (see [101] for a discussion of the range of Hopi uses for these plants). Our review of currently available soil salinity data suggests that agriculture within the canyon could have supplied local residents with sufficient maize without demanding substantial import from distant sources.

Despite the uncertainties associated with studies of soil salinity, this analytical approach still offers potentially useful insights for understanding the natural and anthropogenic landscape within Chaco Canyon. An interesting direction for future research would be defining the relation of canyon floor soil salinity to increasing wash discharge given the significantly different results further upstream [86]. Another direction would be careful association of soil depths and high salinity levels relative to well-dated episodes of surface stability and associated paleosols, which may be indicative of leaching salts to lower depths from irrigation, assuming that the area can be shown to have been an agricultural field. In this paper, we provide data that indicate that Chaco Canyon soils exhibit similar ranges and patterns of salinity to those found in modern, productive agricultural fields and that the continual, long-distance agricultural provisioning of the local residents was improbable. Given this contextualized reassessment and the presence of documented hydro-engineering features likely associated with ancient irrigation agriculture in the canyon, future research on the nature and source of food for Chacoan populations would be better served by focusing on defining the extents of local production areas and further investigating agricultural practices employed within the canyon.

## Supporting information

S1 Notes S1_Supplemental Notes. Notes related on sample process handling, data interpretation, or relevant but specific details of original publications.
(DOCX)

S1 Table S1_Supplemental table. Compilation of Chaco Canyon soil salinity data broken out by researcher into separate tabs. All relevant conversions are applied with original equations and sources noted.
(XLSX)
