# Supplementary material for: Soil analysis in discussions of agricultural feasibility for ancient civilizations: A critical review and reanalysis of the data and debate from Chaco Canyon, New Mexico
Source: PLoS One. 2018 Jun 14;13(6):e0198290. doi: 10.1371/journal.pone.0198290 (PMC6002086; doi:10.1371/journal.pone.0198290)
Supplement: S1 Notes — Notes related on sample process handling, data interpretation, or relevant but specific details of original publications. (DOCX) [file pone.0198290.s001.docx]

**Supplemental Notes**

**A** Values are typically reported in dS/m (deciSeimens per meter), mS/cm (milliSiemens per centimeter), or mmho(s)/cm (millimho(s) per centimeter). All of which are equivalent: 1 dS/m = 1 mS/cm = 1 mmho/cm. All conductivity values in this paper are presented in dS/m.

**B** Benson and Berry [1] discussed the range of conductivities in the San Juan floodplain and refer readers to Supplementary Table 1 of Benson et al. [2]. Though conductivities are presented in that table, how these were obtained is not explained, and results are labeled only as conductivity. The only soil solution described in Benson et al. [2], which is included in the supplementary methods, describes a 1:100 soil:1-M acetic acid preparation. This would not produce a solution appropriate for a conductivity measurement relating to soil salinity.

**C** The figure 1.7 dS/m is actually only given in Benson and Berry [1], where it is attributed to Maas and Hoffman [3]. In the sentence following that citation, Benson and Berry [1] cite a value of ~6.5 dS/m as the point at which maize yields would approach zero and attribute it to Maas and Hoffman [3]. However, Maas and Hoffman [3] do not give a specific value, but instead present an equation for salinity induced yield decrease of Y = 100 – B(ECe – Λ) where Y=yield, B=%yield decrease per unit salinity increase (12% for maize), ECe=measured ECe conductivity, and Λ=salinity threshold (1.7 dS/m). Setting Y to 0 yield and solving for conductivity gives an ECe of 10.03 dS/m, not 6.5.

**D** The actual threshold value is not consistent across Benson et al. [4], Benson and Berry [1], and Benson [5,6], though 1.7 dS/m is what the source cited by each actually states. Benson et al. [4] gives an uncited value of 1.8 dS/m, Benson [5] gives a value of “about 1.5 dS/m” citing Ayers (1977), and Benson (2016) gives ~1.5 dS/m citing Ayers [7] and “USDA (2011)”. For clarification, the citation of “USDA (2011)” in Benson [6] is the internet publication of Ayers and Wescot [8] (itself a revision of Ayers and Wescot [9] on the FAO website; both of which are papers for the Irrigation and Drainage Division of the Food and Agriculture Organization of the United Nations.

**E** See note 3 for a discussion of the crop loss value in Maas and Hoffman [3]. Benson [6] states that total maize crop loss would occur at 6.7 dS/m, but the sources cited state EC would have to be 10 dS/m for total loss.

**F** Multiplying by 1.85 is the non-intercept equation presented by Zhang et al. [10], but it is not known if that is the source of this approach. The fact that the Cond. S.P. values are those used in the paper for figures and discussion is not stated, but can be determined by finding the extreme values represented in Benson [11] figures, or taking the means and quartiles within the table.

**G** It is not published that the soil to water ratio is 1:5 in Tankersley et al. [12], but this is noted in Thress [13].

**H** For example, taking the average Na of all sediments in Tankersley [14], 1.28 %, and applying the standard conceptual conversion discussed, the estimated EC_e_ would be 20 dS/m (1.28% = 12,800 ppm. 12,800/640 = 20). It is stressed that doing such a mathematical conversion is wholly invalid because of the difference in tested material and uncertainty in sample mineralogy from ED-XRF data.

**I** Numbers are from map unit descriptions on the Web Soil Survey as checked December 15, 2016. The Notal series is listed, but not described in the San Juan county Soil Survey Manual, whereas in the McKinley County survey the range of salinity values for that series is classified nonsaline to slightly saline (0-4 dS/m) [15,16].

**J** Maize yield formation is terminated by frost, and, depending on degree of yield formation at that point, can reduce final yield 35-50 %. Frost can reduce yield 4-20% during the period the ears are allowed to dry out, depending on the degree of dry matter accumulated. For Chaco Canyon, first frost has the following likelihoods of occurring by: 10% by September 13th, 50% by September 26th, 90% by October 8th. Generally, years with early frosts would have lower total yield while those with late frost would have a slightly higher yield due to better utilization of seasonal moisture and drying time.

**K** The authors do not specifically state measurements are in dS/m, but the values are unrealistically low to be in mS/cm.

**L** The Chaco Research Archive indicates that the bibliography is current through December 2015 and, thus, very recent publications are not included in this calculation. The inclusion of more recent publications is unlikely to drastically alter the overall proportions given the over 3,000 sources currently listed. Publications that referenced agriculture, subsistence, or water management in any way in their titles were counted.

**Supplemental Notes Bibliography**

1. Benson LV, Berry MS. Climate change and cultural response in the Prehistoric American Southwest. Kiva. 2009;75(1):87–117. doi: 10.1179/kiv.2009.75.1.005.
2. Benson LV, Stein JR, Taylor HE. Possible sources of archaeological maize found in Chaco Canyon and Aztec Ruin, New Mexico. J Archaeol Sci. 2009;36(2):387–407.
3. Maas EV, Hoffman GJ. Crop salt tolerance - current assessment. J Irrig Drain Div. 1977;103:115–134.
4. Benson L V, Stein JR, Taylor HE, Friedman R, Windes TC. The agricultural productivity of Chaco Canyon and the source(s) of Pre-Hispanic maize found in Pueblo Bonito. In: Staller JE, Tykon RH, Benz BF, editors. Histories of Maize: Multidisciplinary Approaches to the Prehistory, Linguistics, Biogeography, Domestication, and Evolution of Maize. Boston: Elsevier; 2006. pp. 289-314.
5. Benson LV. Factors controlling Pre-Columbian and Early Historic maize productivity in the American Southwest, Part 1: the Southern Colorado Plateau and Rio Grande Regions. J Archaeol Method Theory. 2011;18(1):1–60. doi: 10.1007/s10816-010-9082-z.
6. Benson L. The Chuska Slope as an agricultural alternative to Chaco Canyon: A rebuttal of Tankersley et al. (2016). J Archaeol Sci Rep. 2017;16:456-471. doi: 10.1016/j.jasrep.2016.10.017
7. Ayers RS. Quality of water for irrigation. J Irrig Drain Div. 1977;103:135-154.
8. Ayers RS, Wescot DW. Water quality for agriculture. Rev. 1, Food & Agriculture Organization of the United Nations. Irrigation and Drainage Paper 29; 1985.
9. Ayers RS, Wescot DW. Water quality for agriculture. Food & Agriculture Organization of the United Nations. Irrigation and Drainage Paper 29; 1976.
10. Zhang H, Schroder JL, Pittman JJ, Wang JJ, Payton ME. Soil salinity using saturated paste and 1:1 soil to water extract. Soil Sci Soc Am J. 2005;69:1146-1151. doi: 10.2136/sssaj2004.0267.
11. Benson LV. Factors controlling Pre-Columbian and Early Historic maize productivity in the American Southwest, Part 2: the Chaco Halo, Mesa Verde, Pajarito Plateau/Bandelier, and Zuni Archaeological Regions. J Archaeol Method and Theory. 2011;18(1):61–110. doi: 10.1007/s10816-010-9083-y.
12. Tankersley KB, Dunning NP, Thress J, Owen LA, Huff WD, Fladd SG, et al. Evaluating soil salinity and water management in Chaco Canyon, New Mexico. J Archaeol Sci Rep. 2016;9: 94–104. doi: 10.1016/j.jasrep.2016.07.014.
13. Thress J. Salinity rates and agricultural productivity at Robert’s Great House, Chaco Canyon, New Mexico (Master's Thesis). University of Cincinnati; 2016. Available from: http://rave.ohiolink.edu/etdc/view?acc_num=ucin1458643889.
14. Tankersley KB. Geochemical, economic, and ethnographic approaches to the evaluation of soil, salinity, and water management in Chaco Canyon, New Mexico. J Archaeol Sci Rep. 2017;12:378-383. doi.org/10.1016/j.jasrep.2017.02.016.
15. Soil Survey Staff. Soil survey of San Juan County, New Mexico. Eastern Part. United States Department of Agriculture, Natural Resources Conservation Service; 1980.
16. Soil Survey Staff. Soil survey of McKinley County Area, New Mexico, MicKinley County and Parts of Cibola and San Juan Counties. United States Department of Agriculture, Natural Resources Conservation Service; 2005.
17. Benson LV, Berry MS. Climate change and cultural response in the Prehistoric American Southwest. Kiva. 2009;75(1):87–117. doi: 10.1179/kiv.2009.75.1.005.
